# Assessment of Different Modalities and Their Impact on Patients with Ruptured Intracranial Arteriovenous Malformation Treated in King Abdulaiziz Medical City in Jeddah, Saudi Arabia

**DOI:** 10.7759/cureus.6969

**Published:** 2020-02-12

**Authors:** Fayez D Alshehri, Noor Mail, Fahad Okal, Ahmed Alzahrani, Ahmed Allehyani, Abdulrauf Samkari, Suliman Alghamdi

**Affiliations:** 1 College of Medicine, King Saud Bin Abdulaziz University for Health Sciences, Jeddah, SAU; 2 Radiation Oncology, King Abdulaziz Medical City, Jeddah, SAU

**Keywords:** intracranial arteriovenous malformations, avm, vascular malformation, embolization, radiosurgery

## Abstract

Background

Intracranial arteriovenous malformation (AVM) is a rare congenital disease that is characterized by an abnormal tangle of blood vessels where arteries abnormally shunt into veins with no intervening capillary bed. Several treatment modalities, such as microsurgical removal, embolization, and stereotactic radiosurgery (SRS), are used to treat AVM either solely or in combination. We aimed to assess and compare the effect, morbidity, and mortality outcomes of mono-treatment with embolization and combined treatment for AVM obliteration.

Methodology

This retrospective cohort study was conducted in the National Guard Hospital Jeddah and reviewed all the AVM patients that visited the center between 2008 and 2017. We assessed presenting symptoms at diagnosis and any co-morbidities as the clinical characteristics and the patients’ AVM and Spetzler-Martin grade as the morphological characteristics. Moreover, we performed a three-year follow-up on suitable patients and assessed their outcomes using the modified Rankin Scale. In addition, we performed follow-up imaging on the patients to evaluate AVM obliteration after any of the procedures.

Results

We included 29 patients treated in our hospital (72.4%, males; 27.6%, females; mean age 40 years). About 65% of the patients underwent mono-therapy consisting of one or more embolization sessions while about 34% underwent combined treatment (embolization + surgery or embolization + SRS). We found more cases of complete obliteration among patients who underwent mono-therapy (52.6%) than among those who underwent combined treatment (30%). Patients who underwent mono-therapy showed better outcomes compared to those who underwent combined therapy; however, the difference did not reach statistical significance.

Conclusions

Embolization mono-therapy appears to be more effective with regards to the obliteration rate and outcome compared to combined therapy with either SRS or surgery in patients treated in our center.

## Introduction

Arteriovenous malformation (AVM) is a rare congenital disease that is characterized by an abnormal cluster of blood vessels where arteries directly shunt into veins without an intervening capillary bed [1]. In addition, these abnormal blood vessels lack a muscularis layer that leads to the dilation of the blood vessels. This, along with the high velocity of blood flow, can lead to rupture and bleeding of the blood vessels. There are several types of AVM that can develop in any part of the body; however, intracranial AVM is the most common [1]. AVM is considered as one of the leading causes of non-traumatic intracranial hemorrhage in people aged less than 40 years old. The prevalence of AVM is about 1:100,000 every year in the USA. In addition, approximately 18:100,000 are adult patients [2]. More than one percent of all strokes are caused by AVM, especially at a young age. In 40-50% of AVM patients, hemorrhage is considered the first manifestation. Seizure, headache, and numbness or weakness of any body part are other early signs and symptoms in patients with intracranial AVM. Depending on the location and size of the cluster in the brain, some patients may experience more severe neurological symptoms including paralysis, vision loss, speech difficulties, confusion, and loss of concentration. These symptoms and signs can occur at any age but usually become apparent between the ages of 10 to 40 years [3].

Since AVM is a complex disorder, the existence of a grading system has become essential. Spetzler-Martin grading (SM) is a system that categorizes AVM into five grades depending on the size of nodules, location, and venous drainage [4]. The modified Rankin Scale (mRS) is another grading system that can be used to assess the clinical outcome of AVM [5]. Although the treatment of AVM is still considered controversial, the main three treatment modalities for AVM, which can be used solely or in combination, are surgical removal, embolization, and stereotactic radiosurgery [6].

Microsurgical removal is the first line of treatment for AVM cases associated with intracranial hemorrhage. Some studies have shown that surgical resection of AVM significantly decreases the seizure episodes. Most of the time, this surgery is accompanied by the use of microscope-integrated indocyanine green (ICG) fluorescent angiography. It is a quick, safe, and inexpensive technique that facilitates resection by providing immediate high-resolution identification of surface feeding arteries and draining veins [7].

Embolization is another AVM treatment modality that is employed depending on the patient’s situation. It involves inserting a catheter into the brain via the femoral vein or artery to deliver glue (or another non-reactive liquid adhesive material) to the AVM opening from the venous part. Eventually, the glue blocks the AVM in the brain. Angiography is performed along with catheterization to aid in the visualization of the procedure [8]. Embolization reduces the blood flow to AVM, intra-operative blood loss, and operative time [9]. Some of the complications associated with embolization include early intracranial bleeding, permanent or local neurological defect, transient severe headache, and seizures [9, 10].

Stereotactic radiosurgery (SRS) is used to treat small brain tumors by focusing on a high single dose of radiation [11]. The main advantage of SRS is that it protects the surrounding tissue from destruction. The treatment course of SRS usually lasts from one to three years, and the duration between treatment and obliteration is termed as the latency period. The success rate of SRS depends on factors such as the location and size of the tumor. The chance of being completely cured is increased when the AVM is small and reaches up to 80 percent when its 3 cm or smaller [12]. Some complications associated with SRS include neurological deficits, cranial nerve deficits, seizures, and headaches [13].

A common issue with AVM treatment is that the treatment modality chosen depends on the physician's experience, availability of modalities, and the institution's protocol to be followed. Many institutions follow different protocols for different situations. However, the ideal modality to be first used is still controversial [14]. Locally, another problem with AVM treatment is that very few studies have been conducted in Saudi Arabia. We aimed to present our experience with using embolization alone and a multimodality treatment approach (embolization and either SRS or surgical intervention) in the management of ruptured brain AVMs since 2008. Specifically, we aimed to evaluate the outcome of AVM treatment, in terms of AVM obliteration and morbidity and mortality outcomes, after combined treatment (embolization and either SRS or surgery) and after Onyx® embolization alone.

## Materials and methods

After obtaining institutional review board approval, this retrospective cohort study was conducted at King Abdulaziz Medical City, Princess Norah Oncology Center, and reviewed all AVM patients who visited from 2008 to 2017. The study population included 29 patients selected from electronic medical records and patients' files. The inclusion criteria were patients with ruptured AVM, older than 15 years old, and having been undergone any of the three treatment modalities (surgery, SRS, or embolization). We excluded patients who were younger than 15 years old or who had incomplete medical records. We assessed age at diagnosis and gender as the demographic characteristics and presenting symptoms (e.g., headache, seizure, and sensorimotor defects) as the clinical characteristics. In addition, we analyzed past medical history with hypertension, smoking, coronary artery disease, and diabetes mellitus.

To determine the Spetzler-Martin grade for each patient, we assessed morphological characteristics of AVM including the location, size, adjacent eloquent area, associated aneurysm, venous drainage, and a number of feeding arteries. In addition, we assessed the anatomic location of hemorrhage, hematoma diameter, and whether a surgical evacuation had been performed.

We also assessed post-operative complications that occurred within two weeks of the procedure including minor defects (e.g. seizure, headache, and numbness or weakness) and major defects (e.g., paralysis, vision loss, difficulty in speaking, confusion, and loss of concentration. In the three-year follow-up, we assessed the outcomes of both patients who underwent mono-therapy and combined treatment using the mRS with a grade of 0-2 indicating good outcome and that of 3-6 indicating poor outcome. A patient was considered to have received mono-treatment if he/she had undergone one or more embolization sessions. On the other hand, a patient was considered to have received combined treatment if he/she had undergone embolization plus another treatment modality (surgery or SRS). In addition, we performed follow-up imaging of the patients to evaluate the obliteration rate of the AVM and determine whether there is complete or partial obliteration.

The collected data were analyzed using Statistical Packages for the Social Sciences program (SPSS) version 20 (IBM Inc., Armonk, USA). Quantitative data were presented as mean and standard deviation while qualitative data were presented as percentages. We compared outcomes and AVM obliteration between mono-therapy and combined therapy groups using Chi-square and Fisher's exact tests with a p-value less than 0.05 indicating statistical significance.

## Results

Patients' characteristics 

In this study, 29 eligible patients (mean age 40 years) were treated in our hospital between 2008 and 2017. Among the 29 patients, 21 (72.4%) were males and 8 (27.6%) were females. About 13.8% of the patients were hypertensive, 3.4% were diabetic, 6.9% were smokers, 3.4% were hypertensive and smokers, 13.8% were diabetic and hypertensive, and 3.4% had coronary artery disease (CAD) with diabetes. The remaining 55.2% had no significant risk factors. At the time of diagnosis, 51.7% of the patients presented with headache, 17.2% with seizure, and 31% with sensorimotor defects. More detailed information is presented in Table 1.

**Table 1:** Demographic and clinical characteristics of the patients (n=29) HTN - hypertension; DM - diabetes mellitus; CAD - coronary artery disease

Characteristics	Percentage (number)
Age (in years)	Mean age: 40 years
15-19	10.3% (3)
20-39	37.9% (11)
40-60	41.4% (12)
> 60	10.3% (3)
Gender	
Male	72.4% (21)
Female	27.6% (8)
Risk factors	
HTN	13.8% (4)
Smoking	6.9% (2)
DM	3.4% (1)
No risk factors	55.2% (16)
HTN and smoking	3.4% (1)
HTN and DM	13.8% (4)
CAD and DM	3.4% (1)
Presenting symptoms	
Headache	51.7% (15)
Seizure	17.2% (5)
Sensorimotor defect	31% (9)

AVM characteristics

We found that 72.4% of the AVMs were found in a lobar location and the remaining 27.6% in a deep location. The specific details of the AVM locations and sizes are listed in Table 2. Most of the AVMs (69%) were small, about 27.6% were medium, and only 3.4% were large. Moreover, almost 80% of the AVMs were located in non-eloquent areas while the remaining 20% were located in eloquent brain areas. We found that 93% of the AVMs had less than three feeding arteries while 6.9% had three to five feeding arteries. More than half (69%) of the AVMs had superficial venous drainage while the remaining 31% had deep venous drainage. According to the Spritzer-Martin scale, 34.5% of the AVMs were Grade I, 31% were Grade II, 24.1% were Grade III, and 10.3% were Grade IV. Only one patient presented with associated remote aneurysm. Detailed information is presented in Table 3. 

**Table 2:** Specific locations of the AVM (n=29) AVM - arteriovenous malformation

Specific location	Number of patients	Percentage
Temporal	5	17.2%
Frontal	4	13.8%
Parietal	9	31.0%
Thalamus or basal ganglia	2	6.9%
Occipital	2	6.9%
Cerebellar	4	13.8%
Dural	1	3.4%
Intraventricular	1	3.4%
Insular	1	3.4%
Total	29	100.0%

**Table 3:** Characteristics of AVM (n=29) AVM - arteriovenous malformation

Characteristics	Percentage (number)
AVM location	
Deep	27.6% (8)
Lobar	72.4% (21)
Size	
Small	69% (20)
Medium	27.6% (8)
Large	3.4% (1)
Eloquence of adjacent brain	
Non-eloquent	79.3% (23)
Eloquent	20.7% (6)
Feeding arteries	
Less than 3	93.1% (27)
3-5	6.9% (2)
More than 5	0% (0)
Venous drainage	
Superficial	69% (20)
Deep	31% (9)
Spetzler-Martin grade	
I	34.5% (10)
II	31% (9)
III	24.1% (7)
IV	10.3% (3)

Multi-modality treatment and postoperative complications

About 65% of the patients received mono-therapy consisting of one or more embolization sessions while 34% received combined treatment (embolization + surgery or embolization + SRS). All the patients (100%) underwent embolization as the first procedure. Post-operative complications were assessed within two weeks of the procedure and we found that 75.9% of the patients did not have any significant post-operative complications. Moreover, 6.9% had hemorrhage, 10.3% had minor defects (e.g. seizure, headache, and numbness or weakness), and 6.9% had major defects (e.g. paralysis, vision loss, difficulty in speaking, confusion, and loss of concentration). Approximately 72% of all patients received one or more additional treatment. About 9.5% of the patients underwent surgery with preoperative embolization, 9.5% underwent surgery only, and about 57% underwent embolization as a second procedure. In addition, 9.5% of the patients underwent SRS. Regarding post-operative complications following the second procedure, 3.8% of these patients had major defects, 3.8% has minor defects, and almost 85% did not have any significant post-operative complications. Detailed information is given in Table 4.

**Table 4:** Post-treatment complications (n=29) SRS - stereotactic radiosurgery

Type of treatment	Number of patients (n=29)	Post-operative complications			
First procedure		Hemorrhage	Major	Minor	No complications
Embolization	29 (100%)	2 (6.9%)	2 (6.9%)	3 (10.3%)	22 (75.9%)
Second procedure					
Surgery +preoperative embolization	2 (9.5%)	0	0	0	2 (9.5%)
Surgery only	2 (9.5%)	0	0	0	2 (9.5%)
Embolization	12 (57 %)	0	0	1 (4.7%)	11 (52.3%)
SRS	5 (23.8%)	0	1 (4.7%)	1 (4.7%)	3 (14.2%)

AVM obliteration

**Figure 1:**
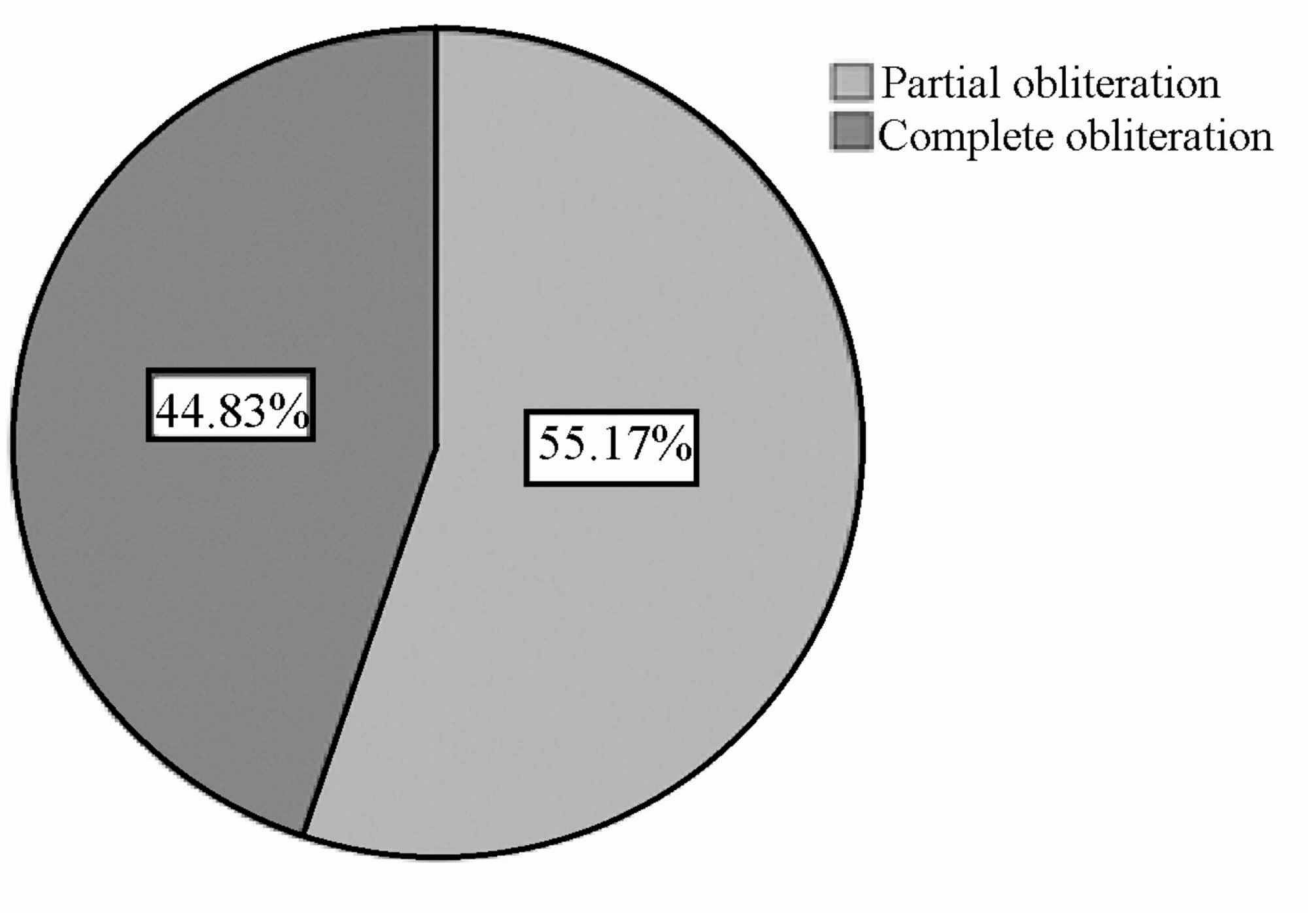
Overall obliteration rate of AVM in all patients after three-years follow-up AVM - arteriovenous malformation

**Figure 2:**
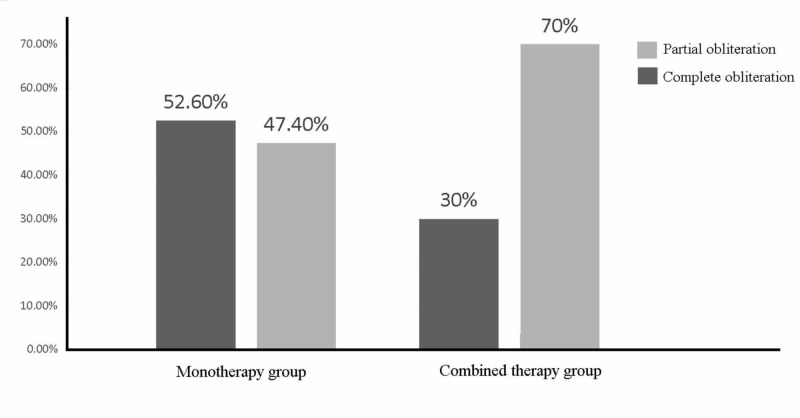
Percentages of partial and complete obliteration in mono-therapy group and combined therapy group

The overall obliteration rate in the patients is shown in Figure 1. There were numerically, but not significantly, more female who showed complete obliteration of the AVM compared to males (p=1.000). There was no significant difference in the obliteration rate between patients aged 40 and above and those aged below 40 (p=0.301). As shown in Figure 2, complete obliteration occurred more in patients who received mono-therapy (52.6%) compared to those that received combined treatment (30%) (p=0.211).

Outcome

**Figure 3:**
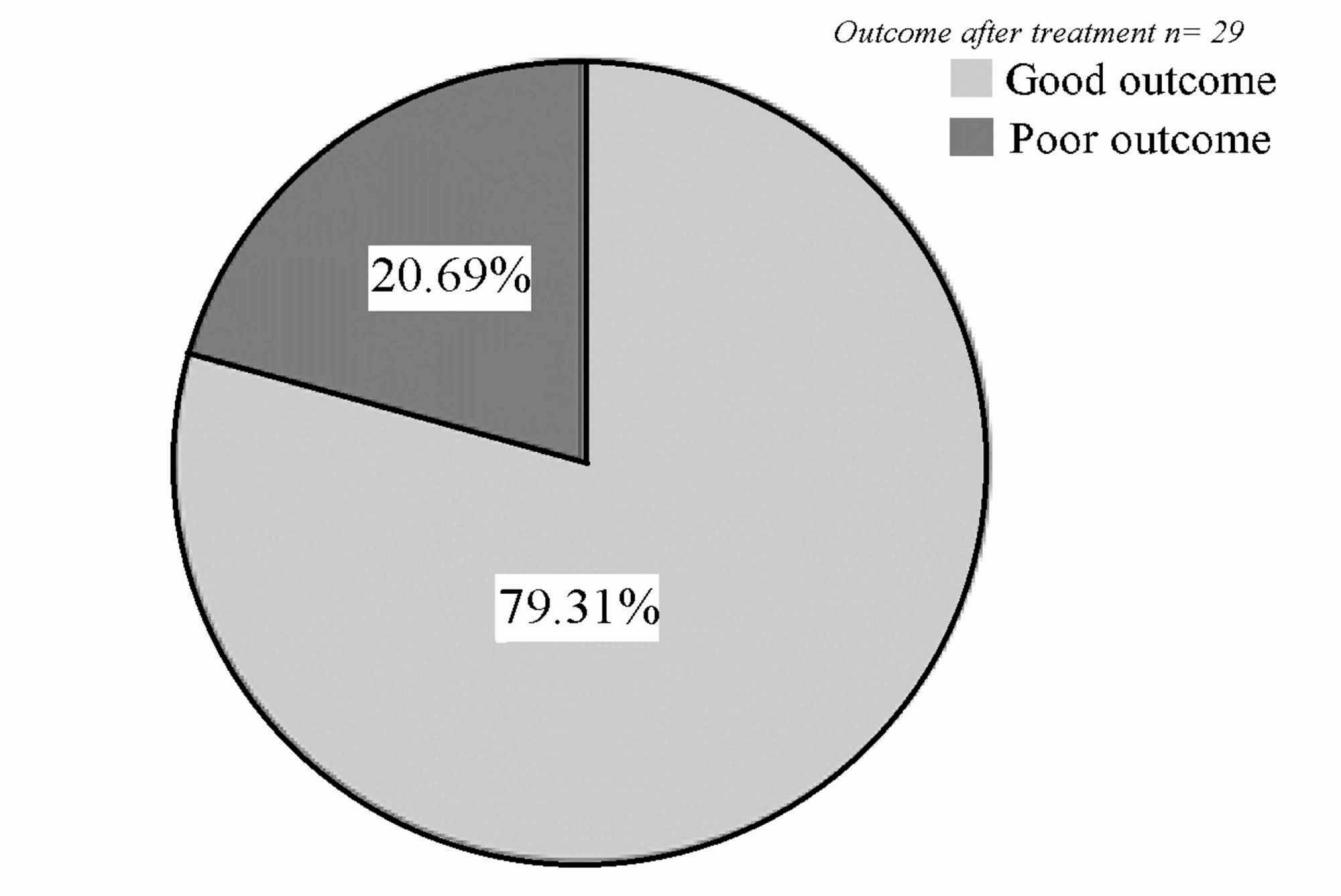
Overall outcomes of the patients after three-years follow-up The modified Rankin Scale grade of 0–2 indicating good outcome, 3–6 indicating poor outcome.

**Figure 4:**
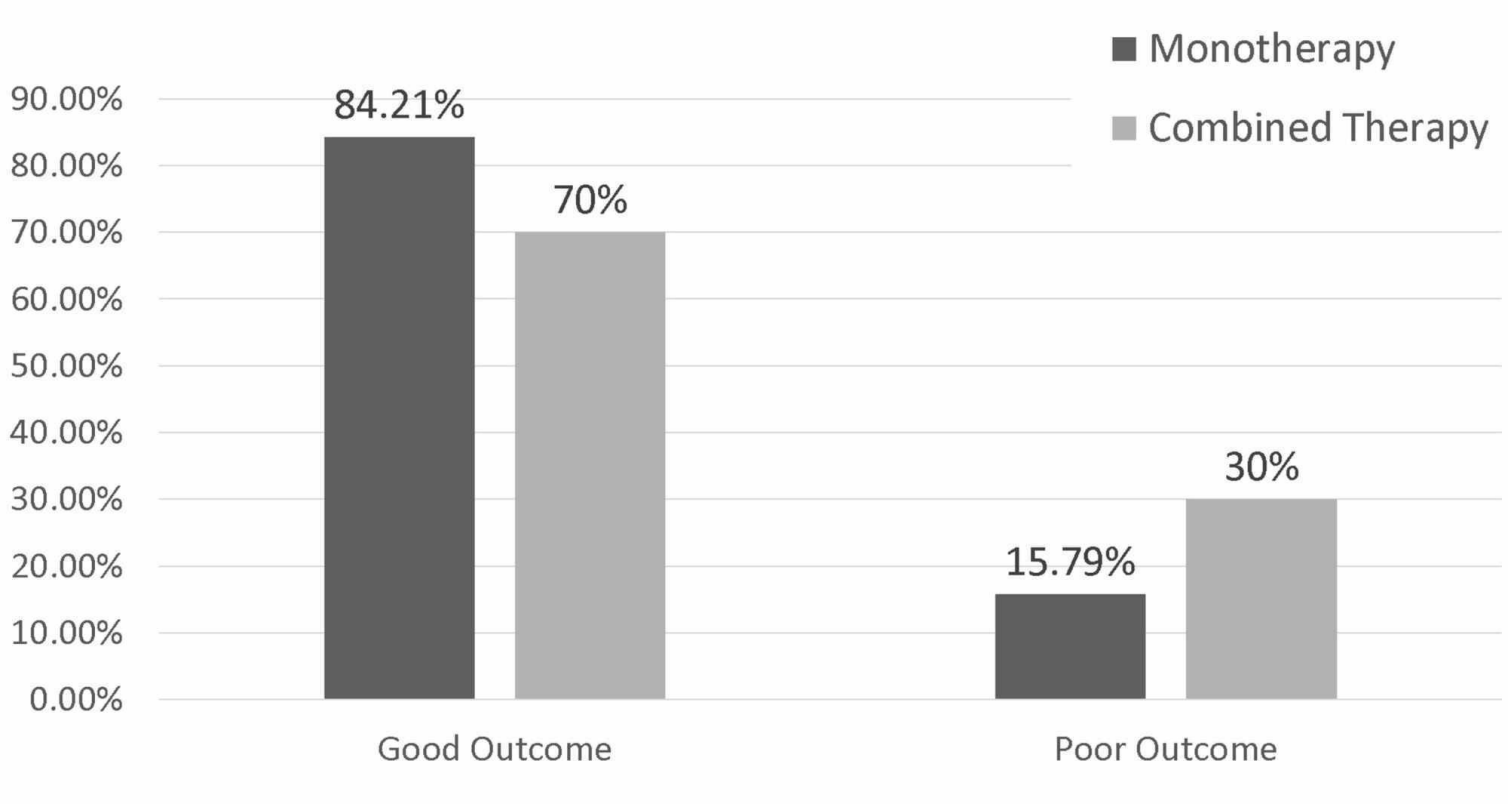
Percentages of good and poor outcome in mono-therapy group and combined therapy groups The modified Rankin Scale grade of 0–2 indicating good outcome, 3–6 indicating poor outcome.

The overall outcomes of the patients are illustrated in Figure 3. Female patients numerically, but not significantly, showed better outcomes compared to male patients (p=0.141). There was no significant difference between the outcomes of patients aged 40 years and above and those younger than 40 years (p=0.651). Patients who underwent mono-therapy showed better outcomes compared to patients who underwent combined therapy; however, it did not reach statistical significance (p=0.632). Detailed information is illustrated in Figure 4.

## Discussion

Embolization is commonly used as the first AVM treatment modality prior to SRS or any other secondary procedure [12]. However, due to various reported outcomes, its use remains controversial. In our study, we compared patients who underwent combined treatment involving embolization and another procedure (SRS or surgery) with those who underwent mono-therapy consisting of embolization only. Our findings on the complete obliteration rate among patients who underwent mono-therapy are almost similar to those of a systematic review that included 15 cohort studies, which reported an obliteration rate of 45.8% [12]. However, we found a slightly less good outcome rate (84.21%) compared to that reported by a case series of 10 patients, which reported a good outcome rate of 90% [13].

Another study reported that combined treatment consisting of embolization and SRS or surgery worked better to reduce the AVM size (52.3% obliteration rate) compared to embolization mono-therapy (25%) [14]. However, in our study, the combined treatment group achieved a lesser obliteration rate (30%). The obliteration rate and outcomes were better in the mono-therapy group than those in the combined therapy group. This could be attributed to the sizes, locations, and grades of the AVM since patients who failed to show AVM obliteration with embolization were treated with SRS or surgery as well. This is one of the primary reasons why combined therapy did not show good outcomes in terms of obliteration [14].

Timing after AVM treatment is critical in determining the outcome of the patients. Pierot et al. in their study performed a series of embolization sessions, followed by combined embolization and SRS treatment on 20 AVM patients between 2003 and 2008. Spetzler-Martin (SM) grades of the AVMs ranged from SM I to II in five patients, SM III to IV in ten patients, and SM V in five patients, with a follow-up period of two to five years after treatment. By five years of follow-up, results showed complete obliteration in 71.4% in low-grade AVMs compared to 50% complete obliteration in high-grade AVMs. Furthermore, complete obliteration rates were higher in five years follow-up compared to a one-year and two-year follow-up period [15]. In our study, the follow-up period for all patients was after three years of the treatment, the reason behind that is to give the SRS treatment enough time to show its optimum effect. These results suggest that timing is an important factor that can influence the outcome and the obliteration rate of the AVM. In addition, it contributes to the controversies between the results observed in various studies and that over time, more patients could achieve successful recovery after the treatment.

## Conclusions

This cohort study did not show any statistically significant difference in the rates of good outcomes or complete obliteration after mono-treatment or combined treatment. Embolization alone appears to be more effective in terms of the obliteration rate and outcome compared to it being combined with SRS and surgery. This may be attributed to the size, location, and grade of the AVMs since most of the complicated cases were treated with SRS or surgery after embolization. However, there is a need for multi-center studies with large sample sizes to attain statistical significance.
